# Commissioning of a double‐focused micro multileaf collimator (μMLC)

**DOI:** 10.1120/jacmp.v11i2.3131

**Published:** 2010-04-16

**Authors:** Marcus Fischer, Manuel Todorovic, Eva Drud, Florian Cremers

**Affiliations:** ^1^ Department of Radiotherapy and Radio‐Oncology, Center for Oncology University Medical Center Hamburg‐Eppendorf Hamburg Germany

**Keywords:** μMLC, IMRT, dosimetry, MLC transmission, MLC penumbra, GafChromic

## Abstract

Double‐focused μMLCs are able to create fields with steeper dose gradients at the field edges and are, therefore, an advancement in delivering stereotactic treatments. A double‐focused μMLC has been installed at a Siemens Primus linear accelerator (linac) as a first research installation in Europe. The basic dosimetric parameters, such as leakage, output factors, depth‐dose curves and penumbra, have been measured in 6 and 15 MV‐mode by use of radiochromic films (GafChromic EBT), ionization chambers and our solid water QA‐phantom (Easy Cube). The leakage between the leaves is minimal and lower than that of most commercially available MLCs. Therefore, the field size of the linac can be kept constant while the leaves of the μMLC are creating different aperture shapes. Percentage depth doses (PDDs) generated by the double‐focused μMLC are equal to depth‐dose curves of the original linac. That means the μMLC affects only the off‐axis ratio (OAR). Based on the fact that the μMLC is double‐focused and the source‐to‐collimator distance is larger, the penumbra is sharper than that for fields defined by the original linac MLC. The mechanical and dosimetric investigations show the benefit of the double‐focused μMLC attached to a Siemens Primus linear accelerator.

PACS number: 87.10.+e

## I. INTRODUCTION

Multileaf collimators are currently regarded as the state‐of‐the‐art device for producing irregularly shaped radiation fields. Due to individually movable leaves fields of high complexity can be generated. A multileaf collimator (MLC) is integrated in almost every modern linac. Considering three‐dimensional conformal radiotherapy, leaf widths of 1–1.25 cm projected to the isocenter are commonly used.^(1)^ ^,^ ^(2)^

Concerning stereotactic treatments (stereotactic radiotherapy/radiosurgery) where small lesions are treated with irregular fields, MLCs with smaller dimensions (micro‐multileaf collimators, μMLCs) are sufficient.^(2)^ Today the leaf width of a μMLC is less than 5 mm.

MLC design and its use are now well established. As a more recent development, MLCs in conjunction with modern treatment planning systems are used by physicists and physicians to modulate the intensity of radiation across a field.

The first technique with intensity modulation was the intensity‐modulated radiotherapy (IMRT), but now there are several new advanced techniques like intensity‐modulated arc therapy (IMAT)^(3)^ or arc modulation optimisation algorithm (AMOA),^(4)^ which have to be applied and commissioned. A double‐focused μMLC could be an improvement to deliver these modern techniques because they are very effective at small and concavely shaped lesions. There is a good overview of IMRT in the AAPM Report No. 82.^(5)^ Double‐focused μMLCs allow steeper dose gradients at field edges so that the dose to healthy tissue can be minimized. The advantages of this focusing with respect to dose distributions have been presented by Meeks et al.^(6)^

In this study, we report on dosimetric measurements, initial acceptance testing and commissioning of the μMLC “L'Azzurro”, manufactured by 3D Line. The measurements were principally used to verify that the unique features of the μMLC, such as leaf design, positioning and monitoring, fulfill the high precision demanded in stereotactic radiosurgery applications. Given that μMLCs as an add‐on for stereotactic treatment have a constant source‐to‐μMLC distance, double‐focusing collimators produce a sharper penumbra than single‐focused devices.^(7)^

## II. MATERIALS AND METHODS

### A. μMLC design features

**Figure 1:**
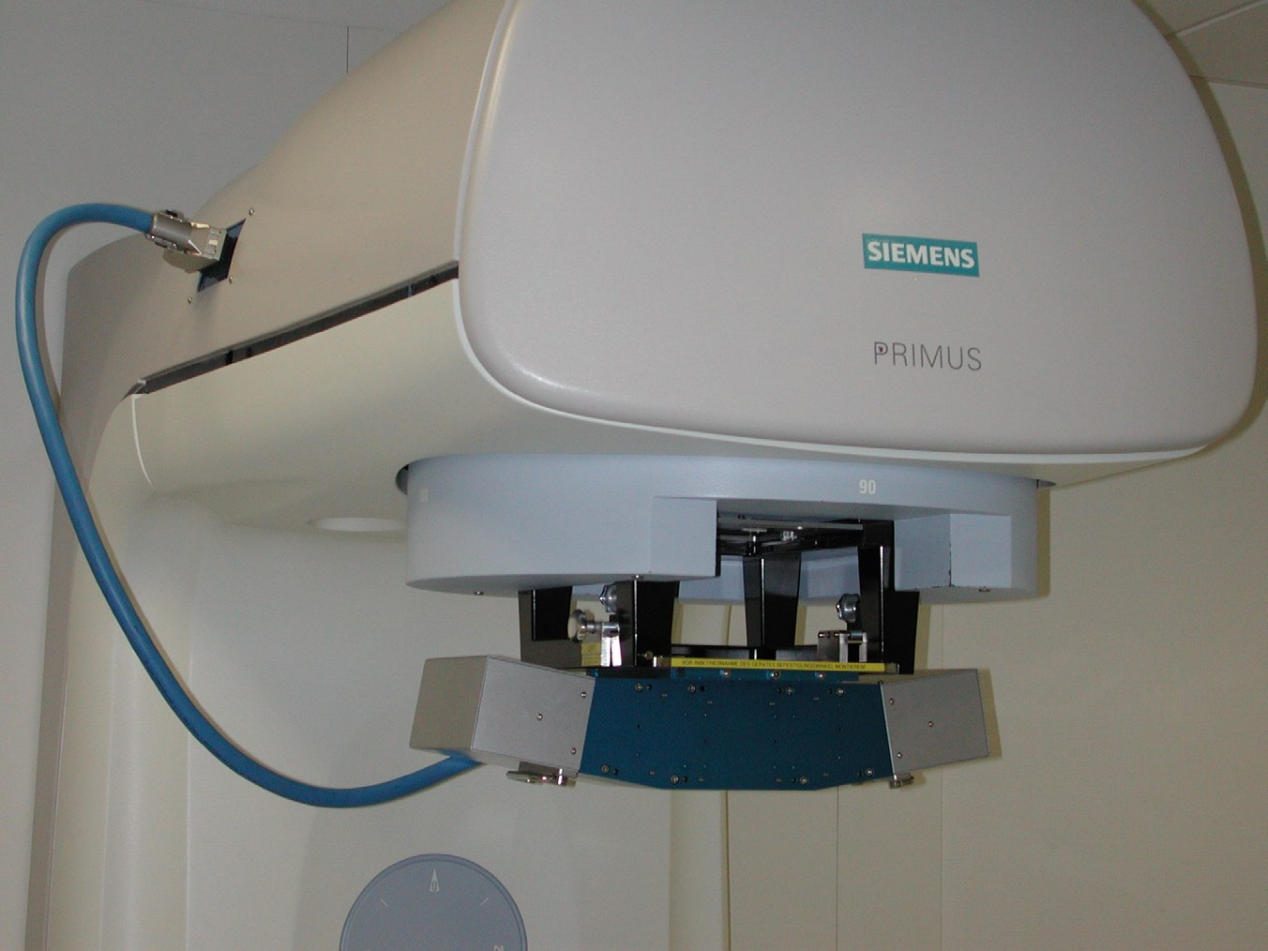
The μMLC attached to a Siemens Primus linear accelerator. The electrical connection between the μMLC and the gantry is established by only one cable.

The dynamic micro‐multileaf collimator (μMLC) provided by 3D Line is part of the DynART system, which consists of the μMLC and its controller as well as an associated planning system called Ergo++. A specific feature is the gantry sensor, which is integrated into the linac's gantry and allows the system to work independently of the type or manufacturer of the linac (i.e., compatible with all accelerators on the market worldwide). The attachment of this μMLC to a Siemens Primus linac (Fig. 1) is one of the first installations in Europe, and the University Medical Center Hamburg‐Eppendorf is the main research department for this collaboration between Siemens and 3D Line.

**Figure 2:**
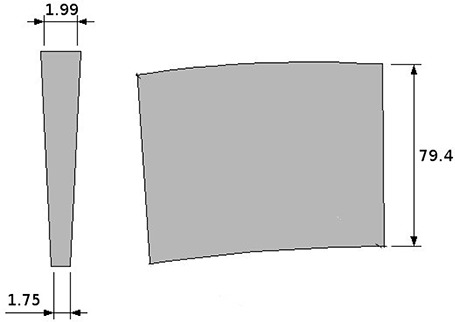
Cross sections and dimensions (in mm) of a single double‐focusing leaf.

The μMLC is double‐focusing and has 24 pairs of 3.27 mm to 2.88 mm (at the lower end) wide tungsten leaves. The leaves have a constant width of 4.7 mm (projected to the isocenter), resulting in a maximum field size of $11.2\times 11\;{\text{cm}}^{2}$. A study of a prototype of the particular MLC investigated in this paper has been previously published by Loi et al.^(8)^ The prototype consisted of only 16 leaf pairs with a constant width of 3.6 mm, making it different from the μMLC used for this study (24 leaf pairs; leaf width varies with distance to center). The leaves move along a curved track so, as the leaves move in and out of the field, the leaf ends are parallel to the beam divergence. In addition, they have a trapezoidal cross section and provide a double‐focused collimation (see Fig. 2). As already mentioned, the μMLC is purported to be usable on any linear accelerator. Naturally each type of accelerator will have a different geometry and this will have an impact on the required divergence. Therefore, leaf edges correspond to the divergence of the beam when the leaf is fully extended (−55mm), centered (0 mm) and fully retracted (+55 mm), which means that they are not at a true focus. The reason of this design is to approximate focusing for a range of source‐to‐MLC distances to accommodate different linac designs. The technical details of this μMLC are described in Table 1.

**Table 1:** Physical characteristics of the μMLC.

Number of leaf‐pairs	24
Leaf height	78 mm
Real leaf width	3.3 mm
Leaf width at isocentre	4.7 mm
Max. field size at isocentre	$11.2\times 11\;{\text{cm}}^{2}$
Leaves repositioning	0.4 mm
Leaf travel over central axis	25 mm
Leaf velocity	>1cm/s
Mass	35 kg

Monitoring of the position of each leaf is realized by two independent methods. Primary positional feedback is derived from the motor shaft's rotation of each leaf, with the number of turns of each motor being related to leaf displacement. Secondary feedback derives from two mechanical brushes physically mounted on and along the longitudinal plane of each leaf. Both methods together ensure the high leaf‐positioning precision (±0.1mm). (See Results section)

For very irregularly‐shaped targets it may be useful to move one or several leaves across the central axis (leaf‐travel‐over‐isocenter). The μMLC has a leaf‐travel‐over‐isocenter of 25 mm. The isocenter clearance is exactly 28.4 cm for a Siemens Primus linear accelerator and is sufficient even for large‐angle non‐coplanar beam orientations.

With the gantry turned to 180°, the μMLC can be manually attached to the accessory mount of the linac by the help of a specially designed trolley. The electrical connection between the dynamic micro‐multileaf collimator (DMLC) and the gantry of the Siemens Primus is based on a single cable, which can be connected very easily. The initialization and calibration procedure is straightforward, and can be completed by two persons within five minutes.

The linac used here has a MLC with 29 leaf pairs integrated in the gantry head. For all measurements, the MLC leaves were set to a constant field size of $11.5\times 11.5\;{\text{cm}}^{2}$ so they would not interfere with the primary beam. Larger fields could affect the electronics and/or radiation could bypass the μMLC. The large aperture was kept for all measurements to prevent an influence on the fields generated by the μMLC.

### B. The treatment planning system (TPS)

For dose calculations, a standard algorithm based upon measurements of tissue maximum ratios (TMR), off‐axis ratios (OAR) and relative output factors is used.^(9)^ ^,^ ^(10)^

The treatment planning system that was used for this study is completely DicomRT‐based. It can import as well as export treatment plans and it can be used with AMOA (Arc Modulation Optimization Algorithm, an additional technique to modulate beams while simultaneously rotating the gantry).

Some of the basic adjustments of the treatment planning system are based on measured output factors (see Results section). Linear accelerators generally show a large dependence of the measured dose rate on the field size if an additional μMLC is used. This is due to various possible scattering events along the way of the photons. As these values are essential parameters for the treatment planning system, the dose rates have to be measured. The field size dependence of the dose rate in the central beam is characterized by relative dose‐rate factors (output factors). They are always related to a field size of $10\times 10\;{\text{cm}}^{2}$ in a standardized depth. Output factors for 6 MV photons were determined. Measurements were performed in a depth of 7.5 cm with 200 MU; 200 MU are equivalent to 2 Gy in reference depth and a $10\times 10\;{\text{cm}}^{2}$ field, as the linac is calibrated this way.

### C. Dosimetry tools

#### C.1 Ionization chamber

Since small field sizes were used, all ionization chamber measurements have been made with a small thimble type chamber (CC03, Wellhofer Dosimetry, Schwarzenbruck, Germany) with air‐equivalent wall material. This chamber is small enough ($0.028\;{\text{cm}}^{3}$ active volume) to minimize volume effects like the lack of lateral electronic equilibrium of very narrow beams^(10)^ ^,^ ^(11)^ and to resolve steep dose gradients.

#### C.2 QA‐Phantom

A solid water phantom, the EASY CUBE (Euromechanics, Schwarzenbruck, Germany), was used for all measurements. The EASY CUBE is a versatile phantom for stereotactic and IMRT validation, made of water‐equivalent material (RW3; polystyrene with 2 % TiO2). It consists of 16 thick slabs $\left(16\times 16{\text{cm}}^{2}\times 1\text{cm}\right)$ housed in walls of 1 cm thickness.

#### C.3 Radiochromic films ‐ GafChromic EBT

In this study we used radiochromic films (GafChromic EBT). These films are commercially available and manufactured by ISP (International Specialty Products, Wayne, NJ). GafChromic EBT films are suitable for doses up to 8 Gy.^(12)^ The absorption spectrum of the radiochromic EBT film has its maximum in the red region of the visible spectrum, at a wavelength of 635 nm. Therefore the analysis of the red channel extracted from a scanned RGB image enhances the response. Its energy response^(12)^ ^,^ ^(13)^ and absorption spectra^(14)^ have been analyzed and discussed elsewhere. For the experiments described here, the films were taken from Lot No. 47261‐06I.

### D. Scanner and Software

For digitizing the irriadiated films we used the flatbed scanner Microtek ScanMaker 8700 (Microtek, Hsinchu, Taiwan). The software we used to analyze the scanned 48‐bit RGB tiff images of the EBT GafChromic films was based on MATLAB 7.0.1. As mentioned above, only the red channel of the RGB tiff image was extracted and analyzed.

The films were individually calibrated by applying a sequence of nine $2\times 2\;{\text{cm}}^{2}$ fields arranged in a 3 by 3 pattern. Each field was irradiated with a different defined amount of monitor units. The analysis of the scanned films was done by self‐written MATLAB routines.^(12)^

### E. Transmission, penumbra and PDD

Transmission was measured with completely closed leaves. The films were positioned perpendicularly to the beam central axis in the EASY CUBE at a depth of 5 cm and a SSD of 100. Films were scanned with the scanner to produce profiles across the closed leaves and at the leaf ends. These scans were normalized to the output measured in an open field with all leaves open. For the open field, a total amount of 150 MU was delivered; for the closed one, a total of 3000 MU was delivered. One hundred MU are equivalent to 1 Gy in reference depth and a $10\times 10\;{\text{cm}}^{2}$ field, as the linac is calibrated this way.

Homogeneous dose distribution in a tumor and sparing of the surrounding healthy tissue can be achieved by using collimating devices like this double‐focusing μMLC. Such a device gives an optimal field shape at each beam direction.^(15)^ Another advantage of such a precise collimation is the independence of the penumbra from the location of the field (off‐axis ratio). Off‐axis ratios were measured with at a depth of 9 cm in the EASY CUBE and a source‐to‐film distance of 100 cm.

Percentage depth dose and off‐axis ratios of field sizes ranging from $0.94\times 0.94\;{\text{cm}}^{2}$ to $10.67\times 10.67\;{\text{cm}}^{2}$ (at a SSD of 92.5) were measured in a water phantom with isocentric setup (Wellhofer Dosimetry). For off‐axis ratios, a depth of 7.5 cm and a SSD of 92.5 were chosen. This SSD is a compromise between the commonly used reference settings (SSD 90 for 15 MV with 10 cm depth and SSD 95 for 6 MV with 5 cm depth). Again, the CC03 ionization chamber was used. As the leaves are designed to match the beam divergence, the penumbra should be independent from the field size. To prove the field size independence, profiles and penumbras were determined for three representative different field sizes.

### F. Mechanical stability

The mass of the μMLC is approximately 35 kg. Although Siemens allows gantry attachments of up to 35 kg, measurements were made to investigate if the installed μMLC causes any additional gantry or collimator sagging when attached to the linac gantry head. This mechanical stability was measured accordingly to a procedure introduced by Winston and Lutz^(16)^ – the so‐called Winston‐Lutz test – which describes the field displacement relative to a 3 mm diameter tungsten ball. This ball was positioned at the radiation isocenter as defined by the treatment room lasers. The gantry was rotated in steps of 45° and, at each angle, the film was exposed to a cross‐shaped field generated by the attached collimator. On the film, the displacement of the field edges relative to the tungsten ball was measured both with and without the μMLC installed.

## III. RESULTS & DISCUSSION

### A. Transmission

**Figure 3:**
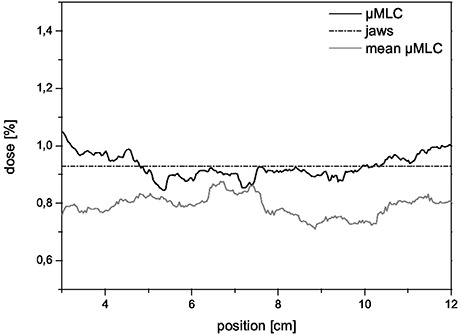
Transmission of the primary collimator and the μMLC measured off‐axis and perpendicular to the field. The transmission through the μMLC averages to 0.93%±0.04%.

Transmission was measured off‐axis and perpendicular to the leaves. Figure 3 plots the ratio of open vs. closed field. The higher profile represents the transmission through the closed leaves of the μMLC, while the primary jaws were closed. The lower profile depicts the transmission of the closed primary jaws while the leaves of the μMLC are open.

The horizontal line represents the mean value for transmission of the μMLC (0.93%±0.04%). This value is fully compliant with the AAPM recommendations,^(18)^ which recommend 2% as a maximum.

For the scan across the central beam axis, the maximum leakage was determined to be 3.4% at the point where the leaves from opposite sites meet at the central axis. If we close the leaves in an off‐axis position of ±40mm, the results for transmission are much better (0.46%). This leakage between leaf ends depends on the proper alignment of the leaves.

In comparison to other published values of leakage, the DMLC has one of the lowest values of all commercially available micro‐multileaf collimators (see Table 2).^(1)^ It is lower because of the slight defocussation of the leaves (see Section II.D)

**Table 2:** Transmission of commercially available μMLCs.

*BrainLAB m3*	*Radionics*	*MRC (Leibinger)*	*DMLC (3D Line)*
<4%	<2%	<1%	<1%

### B. Penumbra and variation of field size

This subsection investigates if the 80–20% penumbra depends on the field size. Several field sizes ranging from $2.83\times 2.83\;\;{\text{cm}}^{2}$ to $9.42\times 9.42\;\;{\text{cm}}^{2}$ were irradiated and evaluated. As an example, a comparison of the penumbras of three different field sizes is presented in Table 3. The profiles were measured in a depth of 5 cm. To evaluate the applied field sizes, the dose profiles in cross‐plane and in‐plane direction were used.

**Table 3:** Mean beam penumbra for three different field sizes.

*Field Size* $\left({\text{cm}}^{2}\right)$	*In‐plane (cm)*	*Cross‐plane (cm)*
2.83×2.83	0.24	0.22
5.65×5.65	0.26	0.25
9.42×9.42	0.25	0.24

The width of the penumbra is nearly independent from the field size or field direction. A look at the penumbra width shows a difference of approximately 2.5% between the $2.83\times 2.83\;\;{\text{cm}}^{2}$ and the $5.65\times 5.65\;\;{\text{cm}}^{2}$ field. This deviation is within the measurement uncertainties of 3%.

The penumbra was 2.4mm±0.15mm and 2.53mm±0.13mm in the direction along the leaf motion and along the side of the leaf, respectively. These values are in good agreement with measurements of similar MLCs, like the BrainLAB m3, which are 2.26mm±0.11mm and 2.31mm±0.11mm.^(17)^

### C. Percentage depth dose (PDD)

**Figure 4:**
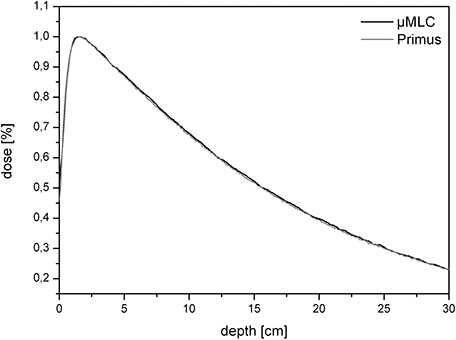
The PDD of a $10\times 10\;{\text{cm}}^{2}$ field size for 6 MV (linac with and without the μMLC). As can be seen, there is no difference between the two curves.

**Figure 5:**
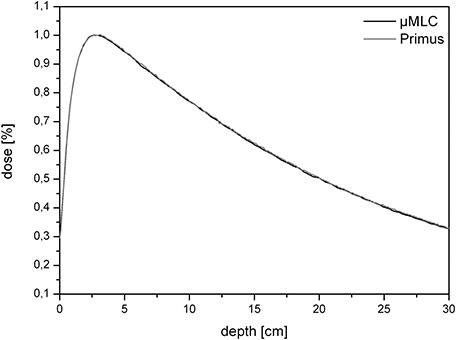
The PDD of a $10\times 10\;{\text{cm}}^{2}$ field size for 15 MV (linac with and without the μMLC). As can be seen, there is no difference between the two curves.

Percentage depth doses with and without the μMLC were measured to detect any differences, whether the μMLC is attached or not. In Figs. 4 and 5, a comparison between two $10\times 10\;{\text{cm}}^{2}$ PDDs for different energies is shown. The field sizes were created by either the integrated MLC or by the additionally installed μMLC. There is almost no visible difference between the two PDDs which means that the μMLC does not affect the depth‐dose characteristics of the linac significantly. The maximal difference amounts to 0.02% and is located before the depth dose maximum in the buildup area. The deviation is considerably smaller than the measurement uncertainty (3%) and is consequently of no statistical or clinical relevance.

### D. Output factors

The ratio between dose measured in the water phantom and the number of applied monitor units depends on the field size (apart from measurement depth and distance to the focus). This field size dependence is characterized by the output factors.

The presented values (Table 4) were measured in a water phantom by an ionization chamber. For 6 MV, we used a depth of 1.5 cm and for 15 MV, a depth of 2.9 cm. Uncertainties are within 2%.

**Table 4:** Output factors for 6 and 15 MV with the μMLC attached.

*Field Size* $\left({\text{cm}}^{2}\right)$	*6 MV*	*15 MV*
0.94×0.94	0.8	0.67
1.88×1.88	0.93	0.88
2.82×2.82	0.95	0.94
3.76×3.76	0.96	0.96
4.71×4.71	0.97	0.97
5.65×5.65	0.98	0.98
6.59×6.59	0.98	0.99
7.53×7.53	0.99	0.99
8.47×8.47	0.99	0.99
9.42×9.42	1	1
10.00×10.00	1	1
10.22×10.22	1	1
10.66×10.66	1	1

Output factors consider the total scattering. This involves the head‐scatter factor (from the linac's head) and the phantom‐scatter factor. Especially for field sizes smaller than 10 cm×10 cm (with which we worked primarily for this paper), the output factor decreases notably. For example, the 6 MV output factor is reduced by 20% when moving from a 10 cm×10 cm to a 0.94 cm×0.94 cm field (1.00 to 0.8). This is, among other things, due to the increasing backscatter on the jaws near to the focus towards the monitor chambers. With the μMLC attached, the primary jaws were set to 11.5 cm×11.5 cm, while the small fields were solely shaped by the μMLC. As a result, the portion of backscatter on the primary jaws is independent of the chosen field size. To take this influence on the output factors into account, new output factors for the case with μMLC have to be measured and used for planning.

### E. Mechanical Stability

**Figure 6:**
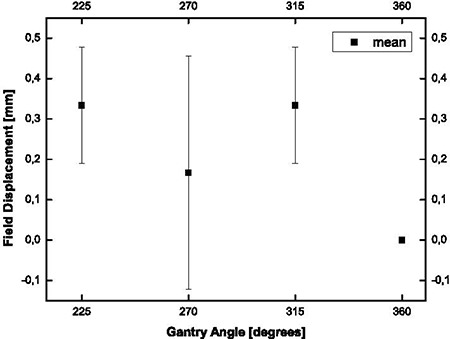
Winston‐Lutz test – field displacement relative to the isocenter as a function of the gantry angle. The field displacement indicates the deviation of the center of the field from the isocenter. The deviations (in mm) for some angles are shown. Collimator angle is always at 90°. Error bars are one standard deviation obtained by four measurements.

At first, to investigate the mechanical stability of the linear accelerator, a Winston‐Lutz‐test of the sole linear accelerator (without the μMLC attached) was performed. This showed no deviation, which means that the linear accelerator itself is correctly aligned and stable in relation to the isocenter. In Fig. 6, the field displacement relative to the tungsten ball as a function of gantry and collimator angle for the linac with the attached μMLC is plotted. The deviations of the isocenter for different gantry angles with the attached μMLC are shown. The criterion for a successful test is a deviation of the isocenter of less than 1 mm. The average of the determined deviation with μMLC was less than 0.5 mm.

### F. Leaf position accuracy

The leaf position accuracy was evaluated by irradiating films with rectangular and irregularly‐formed fields. The films were than scanned (16 scans per film) and the 50% isodose was related to the position of the leaf edge. The value for the target position of the leaf edge was taken from the μMLC control unit. To assess leaf positioning reproducibility, we used the width of the 50% isodose and compared it for different field sizes after repositioning the leaves. Accuracy in the positioning of the leaves was within 0.2 mm.

## IV. CONCLUSIONS

It is important for the regular operation of a μMLC that it be stable and keep the limits necessary for stereotactic treatment. The measurements presented in this paper are helpful for evaluating this. In summary, it can be stated that the μMLC fulfills important criteria for the use for stereotactic irradiation. It accomplishes all of the limit values recommended by AAPM.^(18)^

The dosimetric leaf width has a physically reasonable minimum.^(18)^ The optimum lies at 3 mm. This leaf width is especially important for the use of a micro‐multileaf collimator for intensity‐modulated radiotherapy (IMRT). The μMLC's dosimetric leaf width of 4.7 mm is small enough for the treatment of small lesions.

Due to its special design, the μMLC shows one of the lowest transmission (<1%) of all currently available micro‐multileaf collimators. This enhances the sparing of normal tissue by minimizing unwanted dose exposure which can result in reduced side effects and fewer complications. Again, due to the low transmission of the μMLC, the field size of the linac's integrated MLC can be kept constant while the leaves of the μMLC can shape different apertures.

As we have demonstrated in this paper, the percentage depth doses measured for fields defined by the double‐focused μMLC are equal to the ones from fields defined by the linac MLC. Hence, the mechanic and dosimetric investigations prove the applicability of the double‐focused μMLC attached to a Siemens Primus linear accelerator.

## ACKNOWLEDGEMENTS

The authors would like to thank the German Research Foundation (DFG) for the financial support of this study (project SCHM 1070/24).
